# Data of plant species in permanent plots in a restored coppice-with-standards forest in Northwestern Germany from 1994 to 2013

**DOI:** 10.1016/j.dib.2018.11.046

**Published:** 2018-11-13

**Authors:** Ilka Strubelt, Martin Diekmann, Detlef Griese, Dietmar Zacharias

**Affiliations:** a Applied and Ecological Botany, Faculty 5, University of Applied Sciences Bremen, Neustadtswall 30, 28199 Bremen, Germany; b Vegetation Ecology and Conservation Biology, Institute of Ecology, FB 2, University of Bremen, Leobener Str. 5, 28359 Bremen, Germany; c Gänseweide 5, 38542 Leiferde, Germany

## Abstract

The data presented in this article are related to the research article “Inter-annual variation in species composition and richness after coppicing in a restored coppice-with-standards forest” (Strubelt et al., 2019). The underlying data of that research article are presented here: Monitoring of the vascular plant species composition of 12 permanent plots analysed every year from 1994 till 2002 and again in 2013. For the 2013 survey, data about environmental variables also exist and are included in this data article. The dates of coppicing were recorded for all of these plots, which enabled us to analyse the dynamics of species richness and composition after coppicing on a year to year basis in the above-stated research data article.

**Specifications table**

**Table:** 

Subject area	Ecology
More specific subject area	Forest Ecology and Management, Vegetation Ecology, Biodiversity
Type of data	Excel files
How data were acquired	Sampling of vegetation and environmental variables described in [1]
	Methods: Vegetation (field resurvey on permanent plots); photosynthetically active radiation (LI-COR light meter Model LI-250 and LI-COR Quantum Sensor LI-190; LI-COR, Lincoln, NE, US); soil water content (HH2 Moisture Meter and a Theta Probe Model ML2x); soil pH (standard glass electrode); plant available phosphorus (P); photometric determination by flow injection analysis (FIA Tecator 5012 analyser, 5042 detector; Foss, DK); calcium (Ca), magnesium (Mg) and potassium (K); flame atomic absorption spectrophotometry (AAS Philips PU 9100; Phillips, Amsterdam, NL); total C and N (elemental analyser (HEKAtech Euro EA; HEKAtech, Wegberg, DE)
Data format	Raw data
Experimental factors	12 plots of 20 m × 20 m were analysed with respect to their vascular plant species composition on ten occasions from 1994 to 2002 yearly and again in 2013 (except for one plot that was not sampled in 1994).
	In 2013, soil samples were collected in the plots and analysed for pH, P, Ca, Mg, K, C and N. Photosynthetically active radiation (PAR) and soil water content were also measured in 2013 in every plot.
Experimental features	In each plot the cover of the tree, shrub and herb layers as well as the cover abundance of each species in each layer were recorded according to the Londo (1976) cover-abundance scale [2].
Data source location	The studied area is situated within the 173 ha large CWS forest project Liebenburg, part of the Salzgitter Höhenzug mountains between Liebenburg and Goslar in Germany (N-S 51°58′46.1″-51°58′04.2″N and W-E 10°25′08.5″-10°25′39.0″E, 220–290 m asl).
Data accessibility	Data are available in this article
Related research article	Strubelt, I., Diekmann, M., Griese, D. & Zacharias, D. 2019. Inter-annual variation in species composition and richness after coppicing in a restored coppice-with-standards forest. Forest Ecology and Management 432: 132–139 [1].

**Value of the data**

• This data can be used to compare the inter-annual changes of species richness and composition after coppicing with other coppicing restoration projects.
• This data can be used to compare the effects of coppicing restoration on species richness and composition with other coppicing restoration projects.

## 1 Data

This data article contains three excel sheets:

1. Strubelt et al. DiB 01 Vegetation data: Abundance (Londo cover values) of all vascular plant species in the different layers (tree, shrub, herb) in the 12 permanent plots in the coppice-with-standards forest project Liebenburg investigated in the years 1994–2002 and 2013.
2. Strubelt et al. DiB 02 Environmental data: Environmental parameters of the 12 permanent plots in the coppice-with-standards forest project Liebenburg: Photosynthetic active radiation (PAR), Soil water content (H_2_O), soil pH (pH), contents of the soil nutrients: plant available phosphorus (P), magnesium (Mg), calcium (Ca), potassium (K), C/N ratio (C/N). All parameters were measured in July 2013.
3. Strubelt et al. DiB 03 Coppicing data: Years of 1st and 2nd coppicing of 12 permanent plots in the coppice-with-standards forest project Liebenburg as well as the state of the plot in each year in terms of years after coppicing. NA: No investigation, n.c. = not yet coppiced.

## 2 Experimental design, materials, and methods

A detailed description of the methods can be found in [1]

### 2.1 Sampling of vegetation

12 permanent plots (20 m × 20 m) were analysed with respect to their vascular plant species composition from 1994 to 2002 yearly and again in 2013 (except for one plot that was not sampled in 1994). In each plot, the cover of the tree, shrub and herb layers as well as the cover abundance of each species in each layer was recorded according to the Londo cover-abundance scale [2].

### 2.2 Sampling of environmental variables

In 2013, soil samples were collected in the plots and analysed for pH, P, Ca, Mg, K, C and N. Photosynthetically active radiation (PAR) and soil water content were also measured in 2013 in every plot.
