# Supplementary material for: Data of plant species in permanent plots in a restored coppice-with-standards forest in Northwestern Germany from 1994 to 2013
Source: Data Brief. 2018 Nov 13;24:103461. doi: 10.1016/j.dib.2018.11.046 (PMC6441704; doi:10.1016/j.dib.2018.11.046)
Supplement: Supplementary file 1 — Supplementary material [file mmc1.doc]

Conflict of Interest and Authorship Conformation Form

Please check the following as appropriate:

- All authors have participated in (a) conception and design, or analysis and interpretation of the data; (b) drafting the article or revising it critically for important intellectual content; and (c) approval of the final version.
- This manuscript has not been submitted to, nor is under review at, another journal or other publishing venue.
- The authors have no affiliation with any organization with a direct or indirect financial interest in the subject matter discussed in the manuscript

Author’s name Affiliation

**Authors**:
Ilka Strubelt ^1, 2^, Martin Diekmann ^2^, Detlef Griese ^3^ & Dietmar Zacharias ^1^

**Strubelt, I.** (corresponding author, ilka.strubelt@hs-bremen.de) ^1, 2^

**Diekmann, M.** (mdiekman@uni-bremen.de) ^2^

**Griese, D.** (detlef.griese@t-online.de) ^3^

**Zacharias, D**. (dietmar.zacharias@hs-bremen.de) ^1^

**Affiliations**:

^1^ Applied and Ecological Botany, Faculty 5, University of Applied Sciences Bremen, Neustadtswall 30, 28199 Bremen, Germany,

^2^ Vegetation Ecology and Conservation Biology, Institute of Ecology, FB 2, University of Bremen, Leobener Str. 5, 28359 Bremen, Germany

^3^ Dr. Detlef Griese, Gänseweide 5, 38542 Leiferde, Germany
